# Clinical characteristics of a large familial cohort with Medullary thyroid cancer and germline Cys618Arg *RET* mutation in an Israeli multicenter study

**DOI:** 10.3389/fendo.2023.1268193

**Published:** 2023-10-30

**Authors:** Rachel Chava Rosenblum, Dania Hirsch, Simona Grozinsky-Glasberg, Carlos Benbassat, Uri Yoel, Avraham Ishay, Sagit Zolotov, Gideon Bachar, Ehud Banne, Sigal Levy, Orit Twito

**Affiliations:** ^1^ Endocrine Unit, Edith Wolfson Medical Center, Holon, Israel; ^2^ Sackler Faculty of Medicine, Tel Aviv University, Tel Aviv, Israel; ^3^ Endocrinology, Rabin Medical Center Beilinson Hospital, Petah-Tikva, Israel; ^4^ Neuroendocrine Tumor Unit, Endocrinology & Metabolism Service, Hadassah-Hebrew University Medical Center, Jerusalem, Israel; ^5^ Endocrine Institute, Assaf Harofeh Medical Center, Zerifin, Israel; ^6^ Endocrinology Institute, Soroka University Medical Center, Beer Sheva, Israel; ^7^ Endocrine Unit, Haemek Medical Center, Afula, Israel; ^8^ Rappaport Faculty of Medicine, the Technion, Haifa, Israel; ^9^ Institute of Endocrinology, Diabetes and Metabolism, Rambam Health Care Campus, Haifa, Israel; ^10^ Department of Otorhinolaryngology, Rabin Medical Center, Petah-Tikva, Israel; ^11^ The Rina Mor Genetic Institute, Edith Wolfson Medical Center, Holon, Israel; ^12^ Statistics Education Unit, The Academic College of Tel-Aviv Yaffo, Tel Aviv, Israel

**Keywords:** Medullary thyroid cancer, Cys618Arg RET mutations, RET mutations, Medullary thyroid cancer genetics, MEN2, multiple endocrine neoplasia type 2

## Abstract

**Objective:**

To determine genealogical, clinical and pathological characteristics of a cohort with Cys618Arg mutation from an Israeli multicenter MTC study.

**Methods:**

Retrospective database analysis examining RET mutations and comparing Cys618Arg and Cys634Arg/Thr/Tyr subgroups.

**Results:**

Genetic testing was performed in 131/275 MTC patients (47.6%). *RET* mutations were found in 50/131 (38.2%), including Cys618Arg (28/50 cases,56%), and Cys634Arg/Thr/Tyr (15/50,30%). Through genealogical study, 31 MTC patients were found descendants of one family of Jewish Moroccan descent, accounting for 27/28 patients with documented Cys618Arg mutation and 4 patients without available genetic testing. Familial Cys618Arg cases (n=31) and Cys634Arg/Thr/Tyr cases (n=15, from 6 families) were compared. Although surgical age was similar (25.7 vs 31.3 years, p=0.19), the Cys618Arg group had smaller tumors (8.9mm vs 18.5mm, p=0.004) and lower calcitonin levels (33.9 vs 84.5 X/ULN, p=0.03). Youngest ages at MTC diagnosis were 8 and 3 years in Cys618Arg and Cys634Arg/Thr/Tyr cohorts, respectively. Long-term outcome was similar between groups. The Cys618Arg cohort had lower rates of pheochromocytoma (6.5% vs 53.3%, p=0.001) and primary hyperparathyroidism (3.2% vs 33.3%, p=0.01).

**Conclusion:**

This is the first description of *RET* mutation distribution in Israel. Of 131 tested MTC patients, Cys618Arg was the predominant mutation. To the best of our knowledge, this is the largest cohort of Cys618Arg mutation described. For Cys618Arg and Cys634Arg/Thr/Tyr cohorts, MTC was diagnosed earlier than expected, likely due to familial genetic screening, and MTC outcomes were similar between groups. International studies are necessary to further characterize the clinical features of Cys618 mutations due to their relative rarity.

## Introduction

Medullary thyroid cancer (MTC) is a neoplasia of thyroid C-cells derived from the neural crest. MTC presents as part of a hereditary syndrome in 25% of cases, namely multiple endocrine neoplasia (MEN) type 2 or 3 (previously designated type 2A and type 2 B) or the related entity familial MTC (FMTC) (1), currently considered to represent a variant of MEN2 (2). The MEN2 and MEN3 syndromes are caused by activating germline mutations in the *Rearranged during Transfection (RET)* proto-oncogene on chromosome 10 with autosomal dominant inheritance. The *RET* gene encodes a tyrosine kinase receptor found in cells of neural crest lineage (3). Different mutations lead to various molecular effects and levels of gene activation (3) and a genotype-phenotype correlation has been described (4).

*RET* mutation distribution varies between countries (5). Codon 634 mutations are among the most prevalent in previous European and South American studies (4, 6, 7). The distribution of *RET* mutations in the Israeli population has not been previously described, however a syndrome of MEN2A and Hirschsprung’s disease was reported, with Cys618Arg identified as the causative mutation (8, 9). This mutation was initially described as FMTC (9) and more recently in European, Asian and South American studies as one of the less common causes of MEN2 (4, 7, 10, 11). The syndrome has been described in a Swedish family over multiple generations (12), and as the prevalent mutation in small cohorts from Cyprus and Saudi Arabia (13, 14). Overall, clinical and prognostic data regarding the Cys618Arg mutation is limited to small cohorts such as those described above. Larger studies are necessary for a comprehensive evaluation of this mutation, but are lacking due to its relative infrequency.

American Thyroid Association (ATA) 2015 guidelines have classified *RET* codon mutations into moderate, high and highest risk based on mutation-specific data, and recommend timing of thyroidectomy accordingly. The development and progression from C-cell hyperplasia to node-negative MTC to lymph node involvement and distant metastases is age-related, and varies according to *RET* mutation codon (15, 16). *RET* codon 634 mutations are considered high risk, and thyroidectomy is recommended by 5 years of age (1), while codon 618 mutations are classified as moderate risk, with prophylactic thyroidectomy recommended in childhood or young adulthood depending on serum calcitonin levels (1). The MEN2 associated neoplasms pheochromocytoma and primary hyperparathyroidism (PHPT) are more common in codon 634 mutations (50%, 20-30% respectively) than in codon 618 mutations (10-30%, <10% respectively) (5).

A multicenter Israeli MTC database has been developed (17–19). *RET* mutation screening is reimbursed in Israel for patients with MTC and all patients with a family history of MEN2 in a first-degree relative. We aimed to evaluate the familial connection of a large group with the Cys618Arg mutation with a common ethnic background and to define their clinical characteristics. Patients with codon 634 mutations were used as a comparator for disease characteristics and outcomes. As codon 618 mutations are considered less aggressive than codon 634 mutations, better outcomes were expected in the Cys618Arg cohort.

## Methods

The multicenter Israeli MTC cohort database includes 275 patients diagnosed from 1963-2021 and followed-up in 8 tertiary centers throughout Israel. Study design has been described previously (17). The study was approved by the Institutional Ethics committee of all centers involved: Helsinki committee of Meir Medical Center (0323-16-MMC), Rabin Medical Center Institutional Review Board (0403-12-RMC), Helsinki committee of Assaf Harofeh Medical Center (0072-16-ASF), Helsinki committee of Hadassah Medical Organization (0072-16-HMO), Helsinki committee of the E. Wolfson Medical Center (0143-21-WOMC), Soroka Medical Center ethics (Helsinki) committee (0077-17-SOR), HaEmek Medical Center Helsinki committee (0041-17-EMC) and Rambam Health Care Campus Helsinki Committee (0702-20-RMB). In accordance with Helsinki regulations regarding clinical studies based on chart review, informed consent was waived.

The database was searched retrospectively for results of *RET* mutational analysis, and Familial MTC was defined according to genetic mutation and/or clinical criteria. Patient medical records were reviewed for demographic, pathological and clinical variables. *RET* mutation carriers who did not undergo thyroidectomy, and those who underwent prophylactic thyroidectomy with no discrete tumor on pathology were excluded. Clinico-pathological data included tumor size, measured as largest dimension of the primary tumor, focality (solitary or multifocal, unilateral or bilateral), and extension (intrathyroidal vs. extrathyroidal), lymph node metastases (LNM), and distant metastases (DM) at diagnosis, and other MEN2 related comorbidities. After thyroidectomy, cure was defined as the absence of any biochemical or structural evidence of MTC. Persistent/recurrent disease was defined in the presence of calcitonin levels elevated above the upper limit of the normal range (biochemical disease) and/or the detection of structural findings compatible with loco-regional or distant metastatic lesions (structural disease). Calcitonin levels were defined as a proportion of the upper normal limit for the particular assay, as our data came from various institutions, which used different assays with differing normal ranges. For patients who died during the follow-up period, the cause and date of death and any association with MTC were specified.

**Figure 1:**
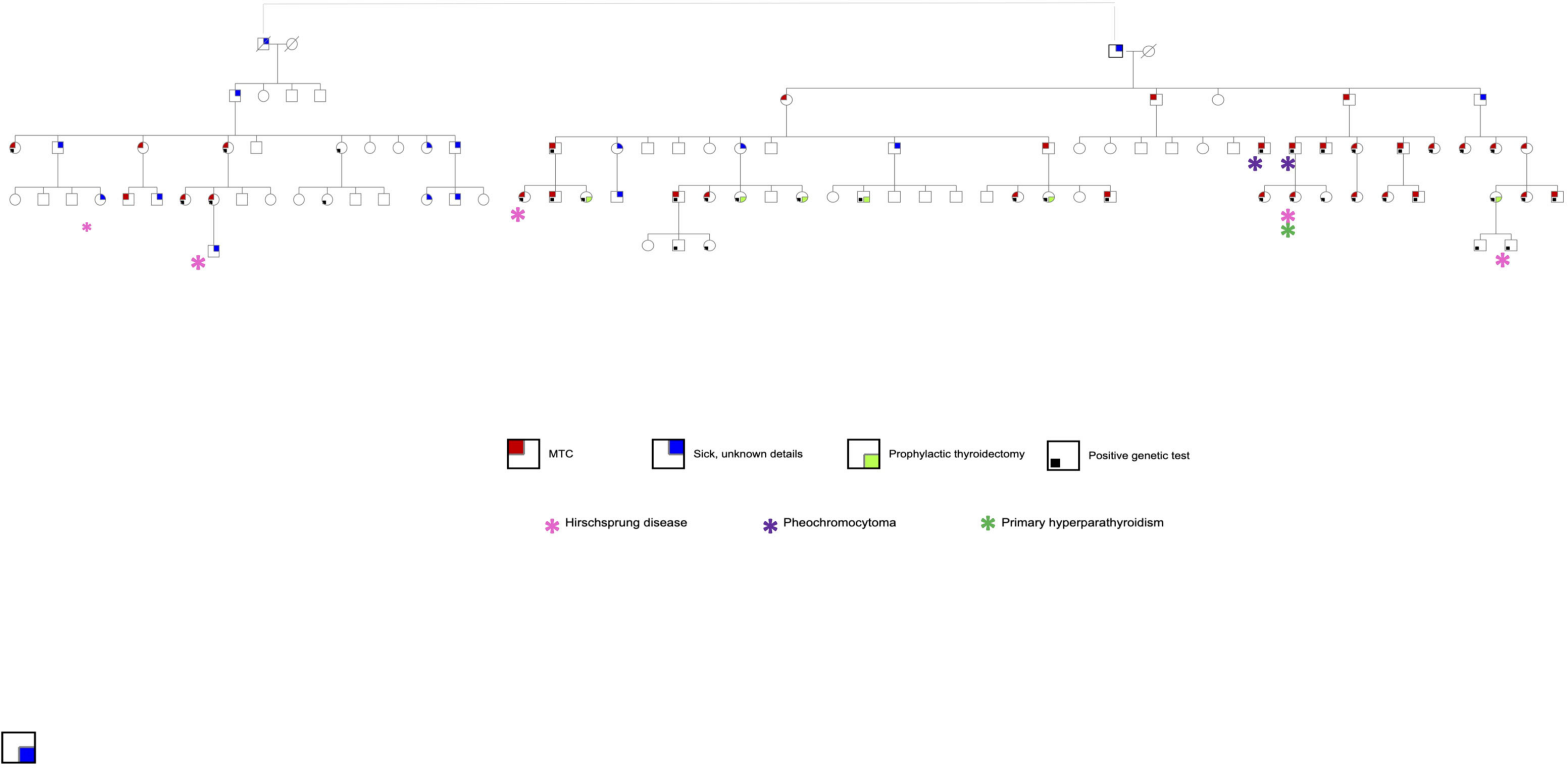
Pedigree of a large family of Jewish Moroccan descent with Cys618Arg mutation.

For the cohort with Cys618Arg mutation, in-depth historical analysis was performed, and a genealogical tree constructed to explore a suspected link between family units. MTC patients who were established first-degree relatives of patients with the known mutation, but with no genetic testing available, were included in this analysis. Family members who had undergone prophylactic surgery with no evidence of tumor, or with suspected MTC who have not undergone surgery, are shown in the genealogical tree (**Figure 1**) although they were not included in the database. Clinical, laboratory and pathological data were retrieved and compared between cohorts with Cys618Arg and Cys634Arg/Thr/Tyr mutations.

Genetic analysis was performed in multiple centers over four decades, and analysis technique differed between centers and according to year of testing. Initially, Sanger sequencing was performed in multiple research laboratories. Later, as repeating variants (founder mutations) were discovered in certain populations and in particular, the Cys618Arg mutation (20), point mutations were offered as the initial workup. Previously, the method for identification of these variants was restriction enzyme, PCR-based. Currently, Sanger sequencing is performed for point mutations, and for multiple mutations – either next generation sequencing or array.

Statistical analyses were performed using SPSS 28 Statistics for Windows. Categorical variables are presented as numbers and percentages, and continuous variables as mean ± standard deviation or median. Comparisons between groups for continuous variables were calculated using the independent samples t-test for non-categorical variables; Mann-Whitney test was also performed and yielded equivalent results. The Chi-square and Fisher’s exact tests were used for categorical variables. Pearson’s correlation coefficient was calculated between continuous variables. Moderation analysis (using Hayes Process model 1) was used to compare the correlations between age and tumor size in each group. Specifically, tumors larger than 40mm were considered outliers and removed from all analyses for tumor size. A p-value of <0.05 was considered statistically significant.

## Results

**Figure 2:**
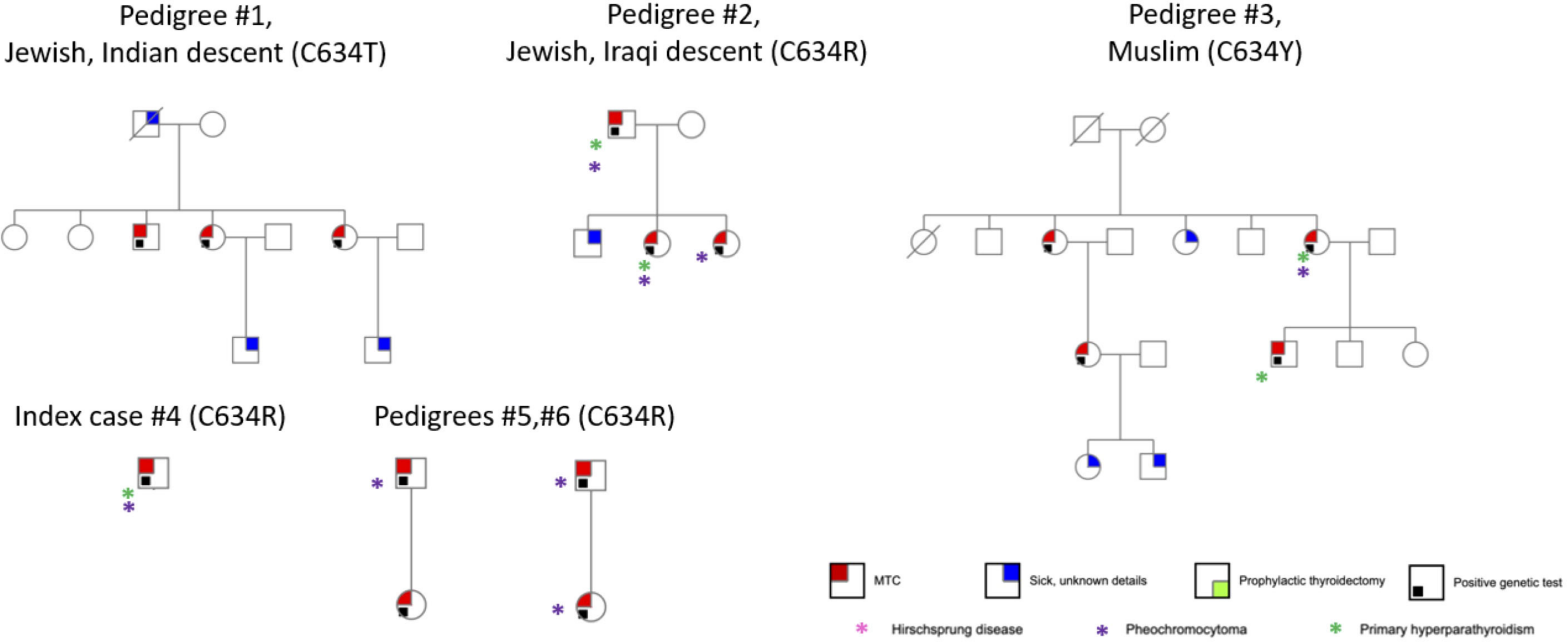
Pedigrees of patients with Cys634Arg/Thr/Tyr mutation.

*RET* mutation analysis performance was documented in 131 MTC cases (47.6%). Of these, 50 (38.2%) had documented germline *RET* mutations and 7 had variants of undetermined significance. Sixty-nine genetic tests were negative, and results were unknown for 5. The distribution of genetic mutations is shown in **Table 1**. The most prevalent mutation was Cys618Arg, accounting for 28/50 cases (56.0%). Through development of a genealogical tree spanning five generations, 27/28 patients were found to belong to a single pedigree, belonging to one large family of Moroccan Jewish origin (**Figure 1**). Patient 28 was of Ukrainian ancestry, with metastatic MTC and pheochromocytoma. His parents, all siblings and children underwent genetic testing and were found to be negative, alluding to a *de novo* mutation with no further inheritance. This patient was excluded from further analysis. Another 4 patients without available genetic testing who belonged to the afore mentioned extended family were included in the analysis. Fifteen patients in the registry had Cys634Arg/Thr/Tyr mutations and were used as a comparator. Of these, 14 belonged to 5 pedigrees of Jewish and Muslim descent (**Figure 2**).

**Table 1:** Distribution of *RET* gene mutations in MTC patients in the Israeli cohort.

Mutation	Number of patients, % (n=50)	Number of pedigrees (families)
Cysteine 618 Arginine (C618R)	28 (56.0%)	2
Cysteine 634 Arginine / Threonine / Tyrosine (C634R/T/Y)	15 (30.0%)	6
Methionine 918 Threonine (M918T)	4 (8.0%)	4
Valine 804 Methionine (V804M)	2 (4.0%)	1
Cysteine 611 Arginine (C611R)	1 (2.0%)	1

**Figure 3:**
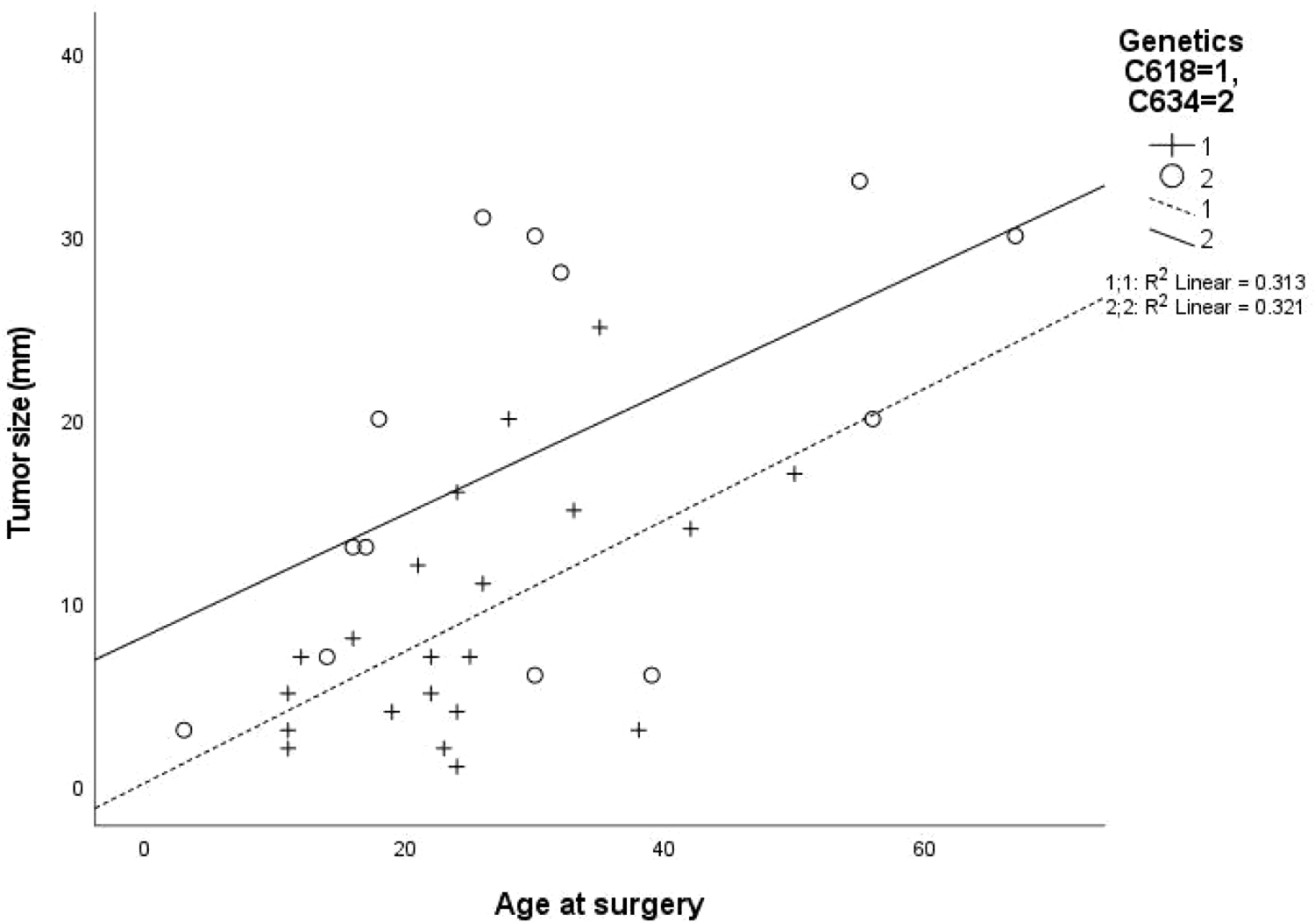
Correlation between patient age at surgery and tumor size for cohorts with Cys618Arg and Cys634Arg/Thr/Tyr mutations.

Clinical, laboratory and pathological characteristics were examined for the two groups (**Table 2**). Age at thyroidectomy did not differ between groups. The youngest MTC patient in the Cys634Arg/Thr/Tyr group was 3 years old; she had no lymph node or distant metastases at diagnosis, and no evidence of recurrence on follow-up. In the Cys618Arg group, the youngest patient was 8 years old at thyroidectomy, and there were 7 cases diagnosed below 18 years of age. While none of these patients developed distant or lymph node metastases at diagnosis, two had persistent/recurrent disease. Total thyroidectomy was performed in all patients with the Cys618Arg mutation with the exception of 2 who underwent hemithyroidectomy, one in 1981 for unknown reasons, and the other in 2020 due to risk of intraoperative recurrent laryngeal nerve injury. There was a trend to perform less lymph node dissections in the Cys618Arg group. The Cys618Arg group had smaller tumors and lower pre-operative calcitonin levels. There were no significant differences in prevalence of lymph node and distant metastases between groups. Mean age at surgery did not differ between groups; however, a significant correlation between age and tumor size was found for each group, and the Cys634 group tended to have larger tumors for age (**Figure 3**). The MEN2-related comorbidities pheochromocytoma and PHPT were significantly more common in the Cys634Arg/Thr/Tyr group; there were only 2 cases of pheochromocytoma in the Cys618Arg group, diagnosed at ages 41 and 50 years, and 1 case of PHPT, diagnosed at 21 years of age. Hirschsprung’s disease was found only in the Cys618Arg cohort, in 2 cases with MTC and 3 more cases within the family (**Table 2**).

**Table 2:** Clinical and pathological features of MTC patients with Cys618Arg and codon 634 mutations in the Israeli cohort.

	C618R (n=31)	C634R/T/Y (n=15)	P value
Age at surgery (years), Mean ± SD, range	25.7 ± 10.9, 8-50 (n=30)	31.3 ± 17.5, 3-67 (n=15)	0.190
Male sex (n,%)	13/31 (41.9%)	6/15 (40%)	0.901
Hemithyroidectomy (n,%)	2/28 (7.1%)	0	
Total thyroidectomy (n,%)	26/28 (92.9%)	12/12 (100%)	1.000
Lymph node dissection (n,%)	13/26 (50%)	10/12 (83.3%)	0.077
Tumor size (mm), Mean ± SD, range	8.9 ± 6.7, 2-40 (n=21)	18.5 ± 11.1, 3-43 (n=13)	**0.004**
Extrathyroidal extension (n,%)	4/19 (21.0%)	2/12 (16.7%)	1.000
Vascular invasion (n,%)	5/18 (27.8%)	2/11 (18.2%)	0.677
Multifocality (n,%)	17/21 (81.0%)	15/15 (100%)	0.125
Bilateral (n,%)	17/22 (77.3%)	15/15 (100%)	0.067
Lymph node metastases (n,%)	6/13 (46.2%)	7/10 (70.0%)	0.402
preoperative calcitonin level*	84.5 ± 201.9 (n=14)	333.9 ± 314.5 (n=9)	**0.030**
Preoperative calcitonin elevated (n,%)	12/14 (85.7%)	9/9 (100%)	0.502
Distant metastases at diagnosis (n,%)	3/26 (11.5%)	1/12 (8.3%)	1.000
Pheochromocytoma (n,%)	2/31 (6.5%)	8/15 (53.3%)	**0.001**
Primary hyperparathyroidism(n,%)	1/31 (3.2%)	5/15 (33.3%)	**0.010**
Hirschsprung’s disease (n,%)	2/21 (6.5%)	0/15 (0%)	

* calcitonin level presented as a proportion of the upper limit of normal range for assay used.

The bold values indicate results with statistically significant differences between groups.

Data comparing long-term outcomes between the Cys618Arg and Cys634Arg/Thr/Tyr groups is portrayed in **Table 3**; no significant differences were found between groups. Persistent disease was found in 45% and 41.7% of patients, respectively (p=0.854). Additional therapy was given to 7 patients in the Cys618Arg group: 4 reoperations, 2 external-beam radiotherapy and 1 tyrosine kinase inhibitors. In the Cys634Arg/Thr/Tyr group one patient underwent further surgery and another underwent I131-MIBG (iodine meta-iodobenzylguanidine) therapy and radiotherapy. Within the Cys618Arg group, 1 patient died due to metastatic MTC within 1 year of surgery, and one more patient died during follow-up of unknown cause. Two patients in the Cys634Arg/Thr/Tyr group died during follow-up; neither death was disease-related.

**Table 3:** Long-term outcome data for MTC patients with codon 618 vs 634 mutations.

	C618R (n=31)	C634R/T/Y (n=15)	P value
Surveillance duration (years), Mean ± SD, range	12.5 ± 12.6, 0-31 (n=31)	12.3 ± 14.0, 0-42 (n=13)	0.974
Disease status one year post-surgery			
Persistent disease (n,%)	9/20 (45.0%)	5/12 (41.7%)	0.854
Biochemical (n,%)	5/8 (62.5%)	4/5 (80.0%)	
Structural (n,%)	3/8 (37.5%)	1/5 (20.0%)	
Mortality (n,%)	1/21 (4.8%)	0	
Disease status at final follow up			
Persistent / recurrent disease (n,%)	11/26 (42.3%)	6/13 (46.2%)	0.819
Biochemical (n,%)	6/11 (54.5%)	5/6 (83.3%)	
Structural / both (n,%)	5/11 (45.4%)	1/6 (16.7%)	
Overall mortality (n,%)	2/31 (6.5%)	2/15 (13.3%)	0.587

Correlation between clinico-pathological factors and outcome were assessed for the Cys618Arg group as shown in **Table 4**. Long-term outcome was affected by tumor size and the presence of lymph node metastases at diagnosis. Although tumor size was correlated with age at surgery, age did not correlate with final outcome. No significant correlation was found between gender, age at thyroidectomy, performance of lymph node dissection, early vs late generation (defined as the earlier three vs later two generations of the cohort as seen in **Figure 1**), maternal vs paternal gene inheritance and outcome. The Cys634Arg/Thr/Tyr group could not be evaluated to this end due to small sample size.

**Table 4:** Factors associated with MTC persistence/recurrence in the Cys618Arg cohort.

Persistence/recurrence 1 year post-operatively			P value	Persistence/recurrence at last visit		P value
	Yes	No		Yes	No	
Age at surgery (years), Mean ± SD, range	29.3 ± 9.9, 15-50 (n=9)	26.7 ± 12.6, 8-49 (n=11)	0.619	28.8 ± 9.0, 11-50 (n=11)	23.8 ± 12.3, 8-49 (n=15)	0.263
Tumor size at surgery (mm), Mean ± SD, range	17.8 ± 5.2, 11-40 (n=5)	7.6 ± 4.9, 2-15 (n=9)	**0.003**	16.0 ± 6.4, 5-40 (n=6)	7.0 ± 4.5, 2-15 (n=12)	**0.003**
Gender						
Male	4/9 (44.4%)	5/9 (55.6%)		5/12 (41.7%)	7/12 (58.3%)	
Female	5/11 (45.5%)	6/11 (54.5%)	1.000	6/14 (42.9%)	8/14 (57.1%)	0.951
Lymph node dissection performed						
Yes	5/9 (55.6%)	4/9 (44.4%)		7/12 (58.3%)	5/12 (41.7%)	
No	3/10 (30.0%)	7/10 (70.0%)	0.370	3/11 (27.3%)	8/11 (72.7%)	0.214
Lymph node metastases						
Yes	5/5 (100%)	0/5 (0%)		6/6 (100%)	0/6 (0%)	
No	0/4 (0%)	4/4 (100%)	**0.008**	1/6 (16.7%)	5/6 (83.3%)	**0.015**
Parental origin of gene						
Maternal	2/7 (28.6%)	5/7 (71.4%)		2/7 (28.6%)	5/7 (71.4%)	
Paternal	7/12 (58.3%)	5/12 (41.7%)	0.350	8/18 (44.4%)	10/18 (55.6%)	0.659
Generation						
Early	5/8 (62.5%)	3/8 (37.5%)		6/10 (60%)	4/10 (40%)	
Late	4/11 (36.4%)	7/11 (63.6%)	0.370	4/15 (26.7%)	11/15 (73.3%)	0.122

The bold values indicate results with statistically significant differences between groups.

## Discussion

In this Israeli multicenter study, Cys618Arg was the most common pathogenic *RET* mutation detected, accounting for more than half of the germline mutations identified in the 131 patients who underwent genetic testing. Genealogical mapping allowed linkage of 31 patients (27/28 patients with documented Cys618Arg mutation) to one large Jewish family who emigrated from Morocco to Israel in the 1950s.

Clinical, pathological and laboratory characteristics of the Cys618Arg and Cys634Arg/Thr/Tyr *RET* mutation groups paralleled known genotype-phenotype correlations of both mutations. Compared with the Cys634Arg/Thr/Tyr group, patients with the Cys618Arg mutation had smaller tumors and lower calcitonin levels at diagnosis; they also tended to be slightly younger at surgery, and to have less extensive surgery. Although our data does not enable us to determine the reason for these differences, we hypothesize that better family surveillance in the Cys618Arg group led to earlier surgeries with prophylactic intent, leading to earlier diagnosis of smaller tumors. We do not have full data regarding surgical intent (whether prophylactic or therapeutic) and did not include patients who underwent curative prophylactic surgery, and therefore cannot discuss the efficacy of prophylactic surgery. The differences could also be potentially attributed, at least in part, to greater aggressiveness of codon 634 mutations.

Importantly, in both groups patients were diagnosed with MTC at ages younger than the ATA guideline cutoffs for prophylactic thyroidectomy. Prophylactic thyroidectomy is currently defined as surgery before MTC development or when MTC remains ‘clinically unapparent’ and confined to the thyroid gland (1). MTC progression has been described as age-related in several *RET* mutations and a large retrospective study described a ‘window of opportunity’ marked off as the youngest age of node-positive MTC described in each mutational risk category (for moderate risk- 15 years and for high risk- 9 years (16)). In our cohort, one patient with codon 634 mutation was diagnosed at 3 years of age; her case was previously published (21). Seven patients in the Cys618Arg cohort were diagnosed in childhood or adolescence (aged 8-16 years). Two patients from the Cys618Arg group diagnosed in childhood had persistent/recurrent disease on follow-up. There was an age-related increase in MTC tumor size for both groups. Although the 2001 MEN syndrome Endocrine Society guidelines described MTC risk level for both codon 618 and 634 mutations as high, and recommended total thyroidectomy by age 5 years for both mutations (22), the 2015 revision of the ATA guidelines for MTC management place Cys618 mutations within the moderate MTC risk category and recommend prophylactic thyroidectomy in childhood or young adulthood depending on serum calcitonin levels (1). Cys634 mutations remain high-risk, and thyroidectomy is recommended by age 5 years (1). Since tumor size and lymph node involvement at diagnosis were the most important factors for disease persistence/recurrence, the timing of surgery is critical in determining outcome.

The incidence of MEN2-related comorbidities with codon 618 mutations has been reported as ≈10-30% for pheochromocytoma and <10% for PHPT (5). In our cohort, pheochromocytoma and PHPT were documented in the Cys618Arg group less frequently than in patients with Cys634Arg/Thr/Tyr mutations, in accordance with the previous literature. Screening for pheochromocytoma and PHPT is recommended from 16 years of age (1) and our data are in line with this recommendation. Hirschsprung’s disease (HD) occurred in the Cys618Arg group only, as previously described (1).

Although the ATA classification of Cys634 mutations as high-risk and of Cys618 mutations as moderate risk, in our study the rates of persistent/recurrent disease did not differ between groups, within the limitation of a relatively small number of cases. Accordingly, a recent large U.S. cohort study did not find any difference in overall survival between high and moderate-risk mutation groups; however, in their cohort, high-risk mutations were associated with earlier disease onset, leading the authors to suggest that mutation risk category may not indicate disease aggressiveness and that future guidelines should classify mutations by disease onset rather than risk level (23). Although our results support this hypothesis, the smaller size of the Israeli cohort may have limited the ability to determine a significant between-group difference in outcome; furthermore, outcome may have been affected by performance of less radical procedures in the Cys618Arg group.

Limitations of our study include its retrospective nature, and the limited sample size, which is inevitable considering the relatively small population of Israel and the rarity of the disease. Our cohort included 8 tertiary medical centers but was not exhaustive of all Israeli hospitals; a recent study based on the Israel National Cancer Registry described 423 cases of MTC diagnosed in Israel between 1980-2009, and supports the assumption that our database includes most of MTC patients in the country (24). Additionally, more than 50% of database patients did not have available genetic test results, allowing for potential selection bias. Our Cys618Arg cohort was one single, extended family, and confounding factors may have influenced results. Variation in clinical expression of *RET* mutations has been previously described between kindreds and within individual families (25). Furthermore, some parts of the genealogical tree depict a pattern of inheritance not typical for autosomal dominant inheritance. This pattern could be explained by consanguineous marriages, or by lack of data regarding family members who were not carriers. Importantly, this study does not distinguish between surgery performed with prophylactic intent and that performed for treatment of preoperatively diagnosed MTC, and full data regarding family members who underwent prophylactic operations with curative outcome was not available.

Looking at previous publications from around the world, Cys618Arg is one of the less-frequent germline mutations in most studies. However, a Saudi cross-sectional study described codon 618 mutations as the most frequent, accounting for 20/43 cases (46%) of hereditary MTC (14). The Cys618Arg mutation was also the most frequent germline cause of MEN2a in a Cypriot study, responsible for 36/40 cases in 9/11 (69.2%) families (13). Interestingly, a small Moroccan study of RET germline mutations in MEN2 patients did not find any cases of Cys618 mutation, although sample size was very limited (26). This could perhaps be explained by the isolated communal living conditions of the Jewish community in Morocco. The geographical variation in MEN2 genotype is hypothesized to be due to genetic drift, and genetic studies have alluded to the presence of common ancestor propagating the genetic mutation via migration and a founder effect (27–30). Genetic analysis would be necessary to determine whether a connection exists between our cohort and others.

In conclusion, we suggest that Cys618Arg is the most common germline RET mutation causing MTC in Israel, and almost all cases were linked to a single large genealogical tree. To the best of our knowledge, this is the largest cohort of Cys618Arg mutation described to date. It is noteworthy that MTC was diagnosed at ages younger than expected in both Cys618Arg and Cys634 mutation groups; prognosis did not differ between groups; and MEN2-related comorbidities were less frequent in the Cys618Arg group. Further global studies are necessary to determine the full spectrum of clinical features and natural history of Cys618 and other uncommon *RET* mutations.

## Data availability statement

The raw data supporting the conclusions of this article will be made available by the authors, without undue reservation.

## Ethics statement

The studies involving humans were approved by ethics boards of each included institution, specified in article. The studies were conducted in accordance with the local legislation and institutional requirements. Written informed consent for participation was not required from the participants or the participants’ legal guardians/next of kin in accordance with the national legislation and institutional requirements.

## Author contributions

RR: Formal analysis, Writing – original draft, Writing – review & editing. DH: Writing – review & editing. SG-G: Writing – review & editing. CB: Writing – review & editing. UY: Writing – review & editing. AI: Writing – review & editing. SZ: Writing – review & editing. GB: Writing – review & editing. EB: Writing – review & editing. SL: Formal analysis, Writing – review & editing. OT: Conceptualization, Writing – review & editing.
